# Extravascular manifestations of Takayasu arteritis: focusing on the features shared with spondyloarthritis

**DOI:** 10.1186/s13075-018-1643-7

**Published:** 2018-07-11

**Authors:** Oh Chan Kwon, Sang-Won Lee, Yong-Beom Park, Ji Seon Oh, Sang Hoon Lee, Seokchan Hong, Chang-Keun Lee, Bin Yoo, Yong-Gil Kim

**Affiliations:** 1 Division of Rheumatology, Department of Internal Medicine, University of Ulsan, College of Medicine, Asan Medical Center, 88 Olympic-ro 43-gil, Songpa-gu, Seoul, 05505 South Korea; 2 Division of Rheumatology, Department of Internal Medicine, Yonsei University College of Medicine, Seoul, South Korea; 3 Clinical Research Center, University of Ulsan College of Medicine, Asan Medical Center, Seoul, South Korea; 4 Department of Radiology and Research Institute of Radiology, University of Ulsan College of Medicine, Asan Medical Center, Seoul, South Korea

**Keywords:** Takayasu arteritis, Extravascular manifestation, Arthritis

## Abstract

**Background:**

Takayasu arteritis (TAK) is a systemic disease characterized by large vessel involvement. Although the vascular characteristics of TAK are well characterized, there is no well-organized study demonstrating the extravascular manifestations of TAK. We aimed to evaluate the characteristics of extravascular manifestations of TAK, and to identify the association between vascular and extravascular manifestations of TAK.

**Methods:**

TAK patients from two independent cohorts between January 2012 and October 2017 were included in the study. Patient characteristics were retrospectively collected from the electronic dataset. The computed tomography scans of all subjects were reviewed to evaluate the pattern of vascular involvement and presence of sacroiliitis. Clinical findings including uveitis, skin lesions, oral ulcers, arthritis, and inflammatory bowel disease (IBD) were reviewed. Logistic regression analysis was performed to evaluate the association between vascular and extravascular manifestations.

**Results:**

For the 268 TAK patients, the mean age at diagnosis was 41.2 ± 14.2 years and 88.1% were female. The extravascular manifestation of TAK was observed in 19.0% of patients, the most common being arthritis including sacroiliitis (11.9%) followed by recurrent oral ulcers (8.6%) and IBD (2.6%). A multivariate logistic regression analysis revealed type IIB vascular involvement (adjusted odds ratio (OR) 2.956, 95% confidence interval (CI) 1.337–6.537, *p* = 0.007) and the erythrocyte sedimentation rate (ESR) (adjusted OR 1.014, 95% CI 1.003–1.025, *p* = 0.012) as significantly associated with the presence of axial and peripheral arthritis.

**Conclusions:**

Extravascular manifestations of TAK were observed in up to one-fifth of patients. The most common extravascular manifestation was arthritis, which was associated with a type IIB vascular involvement pattern and a high ESR.

## Background

Takayasu arteritis (TAK) is a systemic vasculitis mainly involving the aorta and its major branches and can cause occlusive or aneurysmal degeneration [1]. The prevalence of TAK in Japan was estimated at 40 per 1 million of population, with a female predominance [2]. The prevalence of TAK in European studies is lower than that in Japan, being 4.7–13.2 per 1 million of population [3–5].

Despite this rarity, based on a number of previous studies [1, 6–11], knowledge regarding TAK has much improved since its first report in Japan in 1908 [12]. In particular, the vascular manifestations of TAK have been well reported in previous studies [1, 6–11]. Although there might be some discrepancies among different ethnic populations, the largest scale observational study conducted in Japan [1] reported that local symptoms and findings attributable to vascular involvement were most commonly observed in the cervicobrachial area. Accordingly, the majority of the patients exhibited vascular involvements of the aortic arch or its major branches, which supply the cervicobrachial area. Furthermore, the most common type of angiographic involvement according to Hata’s classification [13] was type I (branches of the aortic arch).

Although the vascular manifestations of TAK are well known, the extravascular manifestations of TAK remain unclear. Some recent studies have addressed spondyloarthritis (SpA) [14, 15] and inflammatory bowel disease (IBD) [16, 17] in TAK patients. However, only sporadic case reports regarding other extravascular manifestations such as erythema nodosum [18–20] and uveitis [21, 22] have been reported. Moreover, peripheral arthritis has occasionally been observed in TAK patients, but data concerning these manifestations are not well established. Therefore, we aimed to identify the characteristics of extravascular manifestations of TAK. Furthermore, we evaluated the association between vascular and extravascular manifestations of TAK.

## Methods

### Study population

Two independent cohorts from two tertiary referral hospitals in South Korea were included. All study subjects were diagnosed with TAK and encoded M314 according to the International Statistical Classification of Diseases and Related Health Problems, Tenth Revision (ICD-10) between January 2012 and October 2017. All patients met the 1990 American College of Rheumatology (ACR) classification criteria for TAK [23]. Computed tomography (CT) scans encompassing the entire aorta and its branches as a diagnostic evaluation were present for all patients. Patients who only underwent localized imaging work-up such as carotid duplex sonography or neck angiography were excluded.

Electronic medical records of the study subjects were reviewed, and information pertaining to age, sex, the presence of hypertension (HTN), clinical symptoms and signs, laboratory data at initial presentation including erythrocyte sedimentation rate (ESR) and C-reactive protein (CRP), and imaging data were collected. Regarding clinical symptoms and signs, those attributable to vascular involvement such as claudication, decreased pulse, and blood pressure difference > 10 mmHg between arms, bruit, and carotidynia were reviewed. The CT scans were utilized to assess the distribution of vascular lesions and categorize them according to Hata’s classification [13].

This study was approved by the Institutional Review Board of Asan Medical Center in Seoul, South Korea (IRB No. 2017-0857). The requirement for informed consent was waived due to the retrospective nature of the study.

### Extravascular manifestations

The presence of peripheral arthritis, axial arthritis (sacroiliitis), recurrent oral ulcers, erythema nodosum, IBD (Crohn’s disease (CD) and ulcerative colitis (UC)), and uveitis were determined from the electronic medical records. A radiologist confirmed the presence of sacroiliitis from the CT scans that were taken for the diagnostic work-up of TAK.

For patients with peripheral arthritis, the number and distribution of joint involvements and positivity of rheumatoid factor (RF), anti-cyclic citrullinated peptide (anti-CCP) antibody, and antinuclear antibody (ANA) (if the laboratory test was performed) were reviewed. The number of joint involvements was classified as monoarthritis (1 joint involved), oligoarthritis (2–3 joints involved), and polyarthritis (≥4 joints involved). The distribution of joint involvement was assessed by the size (small joints, large joints, or both), symmetry, and location (upper extremities, lower extremities, or both) of the joints involved. For patients with sacroiliitis, positivity of HLA-B27 (if the laboratory test was performed) and whether they fulfilled the radiologic 1984 modified New York criteria [24] were reviewed.

### Statistical analysis

We described both vascular and extravascular manifestations. Continuous variables were expressed as the mean ± standard deviation (SD) and median (interquartile range (IQR)) for a normal and a non-normal distribution, respectively. Categorical variables were expressed as number (%). To evaluate the association between vascular and extravascular manifestations, we performed logistic regression analysis. Univariate analysis was performed for each variable, and variables determined statistically significant (*p* < 0.05) were included in a multivariate analysis.

## Results

### Baseline characteristics

During the study period, 268 TAK patients (185 and 83 from each cohort, respectively) with the ICD-10 code M314 who met the 1990 ACR classification criteria for TAK [23] were identified.

The mean age of the study population was 41.2 ± 14.2 years. The patients were predominantly female (88.1%). A blood pressure difference of more than 10 mmHg between both arms, decreased pulse, bruit, HTN, claudication, and carotidynia were observed in 84.1%, 56.7%, 45.8%, 44.8%, 33.9%, and 14.4% patients, respectively. The median ESR result was 24.0 mm/h (IQR 13.0–46.0), and the median CRP serum concentration was 0.17 mg/dl (IQR 0.10–0.70) (Table 1).

**Table 1:** Baseline characteristics of the 268 Takayasu arteritis patients

Characteristic	*N* = 268
Age (years), mean (± SD)	41.2 (± 14.2)
Female	236 (88.1%)
Hypertension	120 (44.8%)
Claudication (*N*^a^ = 218)	74 (33.9%)
Decreased pulse (*N*^a^ = 203)	115 (56.7%)
Blood pressure difference > 10 mmHg (*N*^a^ = 239)	201 (84.1%)
Bruit (*N*^a^ = 203)	93 (45.8%)
Carotidynia (*N*^a^ = 201)	29 (14.4%)
ESR (mm/h), median (IQR)	24.0 (13.0–46.0)
CRP (mg/dl), median (IQR)	0.17 (0.10–0.70)
Vessel involvement	
Common carotid artery	176 (65.7%)
Subclavian artery	170 (63.4%)
Ascending aorta	84 (31.3%)
Aortic arch	100 (37.3%)
Descending aorta	151 (56.3%)
Abdominal aorta	124 (46.3%)
Renal artery	54 (20.1%)
Iliac artery	30 (11.2%)
Pulmonary artery	30 (11.2%)
Hata’s classification	
Type I	54 (20.1%)
Type IIA	23 (8.6%)
Type IIB	55 (20.5%)
Type III	10 (3.7%)
Type IV	6 (2.2%)
Type V	120 (44.8%)

Data presented as *n* (%) unless stated otherwise

*SD* standard deviation, *ESR* erythrocyte sedimentation rate, *IQR* interquartile range, *CRP* C-reactive protein

^a^Patients with missing data excluded

### Patterns of vessel involvement

The most frequently involved vessel was the common carotid artery (65.7%), followed by the subclavian artery (63.4%) and the descending aorta (56.3%). The least involved vessels were the iliac and pulmonary arteries (both 11.2%). The most common pattern of vessel involvement according to Hata’s classification [13] was type V (44.8%) (Table 1).

### Extravascular manifestations

Of the 268 patients, 19.0% displayed at least one extravascular manifestation, the most common being arthritis, followed by recurrent oral ulcers, IBD, and erythema nodosum (11.9%, 8.6%, 2.6%, and 1.5%. respectively). Uveitis (unilateral anterior uveitis) was infrequently detected (0.7%). The 32 arthritic patients comprised 19 with sacroiliitis and 16 with peripheral arthritis (Table 2).

**Table 2:** Extravascular manifestations of Takayasu arteritis

Manifestation	*N* = 268
At least one extravascular manifestation present	51 (19.0%)
Arthritis	32 (11.9%)
Axial arthritis (sacroiliitis)	19 (7.1%)
Peripheral arthritis	16 (6.0%)
Recurrent oral ulcers	23 (8.6%)
Inflammatory bowel disease	7 (2.6%)
Crohn’s disease	4 (1.5%)
Ulcerative colitis	3 (1.1%)
Erythema nodosum	4 (1.5%)
Uveitis^a^	2 (0.7%)

Data presented as *n* (%)

^a^Both patients had unilateral anterior uveitis

**Fig. 1:**
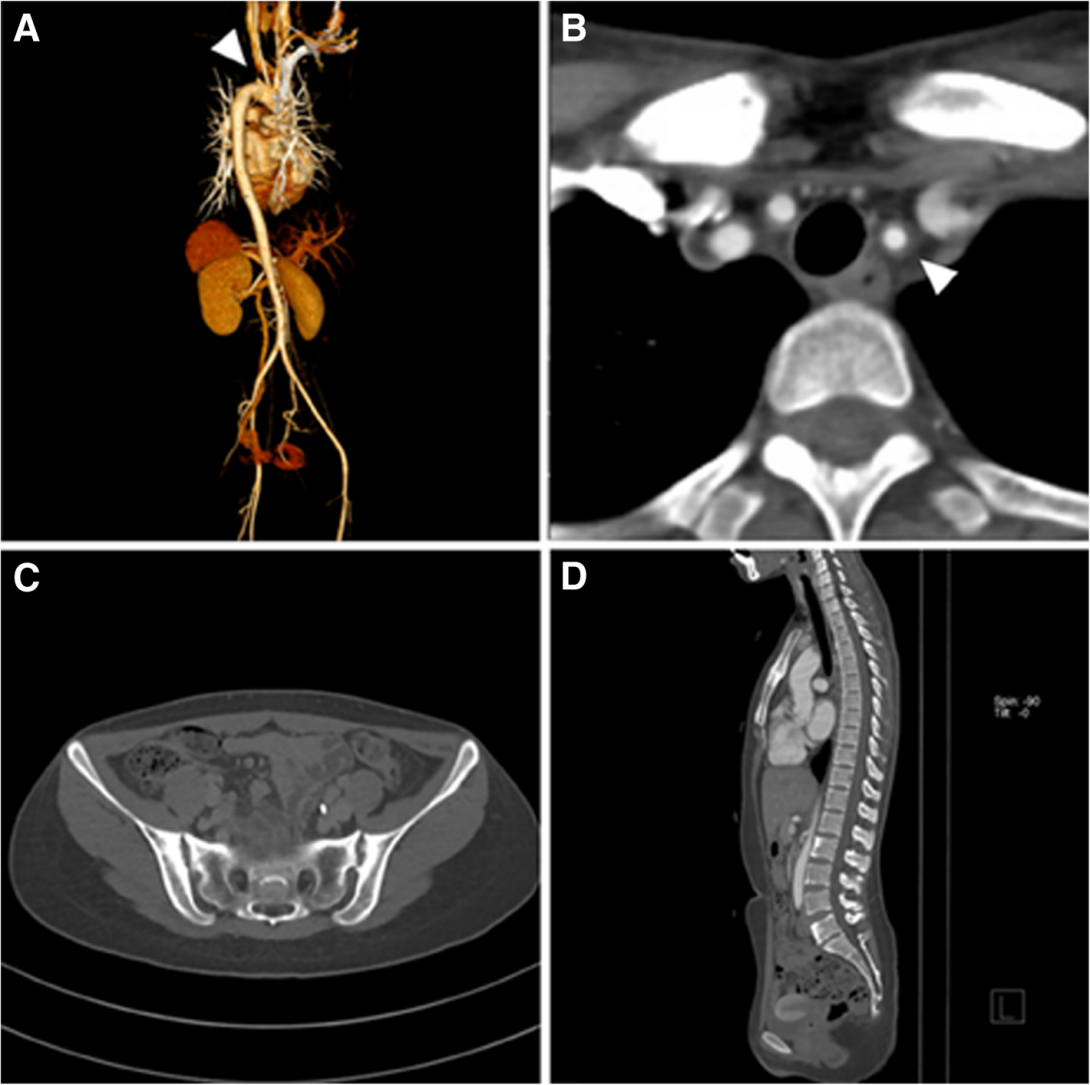
Computed tomography (CT) images of Takayasu arteritis (TAK) patient who exhibited sacroiliitis as an extravascular manifestation. Female patient, 29 years old, negative for HLA-B27. **a** Complete occlusion in left subclavian artery (arrowhead). **b** Concentric wall thickening in left common carotid artery (arrowhead). **c** Sclerosis in both sacroiliac joints and erosion with joint space widening suggesting grade III sacroiliitis. **d** Vertebral corner inflammations, especially in lumbar spines (L2 and L5)

As shown in Table 3, of the 19 patients with sacroiliitis, the majority were female (89.5%) and HLA-B27 negative (85.7%). The 1984 modified New York criteria [24] were met by 68.4% of these patients, and 15.8% exhibited peripheral arthritis. Enthesitis, IBD, and uveitis were present in one patient each. While type V vessel involvement was the most common in the total study population, type IIB was most frequently observed in the patients with sacroiliitis (36.8%). Representative images of a TAK patient with sacroiliitis are presented in Fig. 1.

**Table 3:** Characteristics of the 19 Takayasu arteritis patients with axial arthritis (sacroiliitis)

Characteristic	*N* = 19
Age (years), mean (± SD)	38.9 (± 14.9)
Sex	
Male	2 (10.5%)
Female	17 (89.5%)
HLA-B27 (*N*^a^ = 7)	1 (14.3%)
Sacroiliitis meeting 1984 modified New York criteria^b^ for AS	13 (68.4%)
Peripheral arthritis	3 (15.8%)
Enthesitis	1 (5.3%)
Inflammatory bowel disease	1 (5.3%)
Uveitis	1 (5.3%)
Hata’s classification	
Type I	3 (15.8%)
Type IIA	2 (10.5%)
Type IIB	7 (36.8%)
Type III	1 (5.3%)
Type IV	0 (0.0%)
Type V	6 (31.6%)

Data presented as *n* (%) unless stated otherwise

*SD* standard deviation, *AS* ankylosing spondylitis

^a^Patients with missing data excluded

^b^Radiologic criteria of the 1984 modified New York criteria [24]: sacroiliitis grade ≥ 2 bilaterally or grade 3–4 unilaterally

The characteristics of the 16 patients with peripheral arthritis are presented in Table 4. Monoarthritis, oligoarthritis, and polyarthritis were observed in 37.5%, 43.8%, and 18.8% of patients, respectively. Regarding joint distribution, large joint involvement was most common (62.5%), and the shoulder joint most frequently affected. The majority of patients exhibited asymmetric joint involvement (87.5%). The locations of involved joints were approximately evenly distributed between the upper extremities, the lower extremities, and both the upper and lower extremities together. RF was positive in 9.1%, and none of the patients were positive for anti-CCP antibody or ANA. The most common vessel involvement was type IIB (50%), which is concordant with that of the study subjects with sacroiliitis.

**Table 4:** Characteristics of the 16 Takayasu arteritis patients with peripheral arthritis

Characteristic	*N* = 16
Age (years), mean (± SD)	41.6 (± 14.7)
Sex	
Male	2 (12.5%)
Female	14 (87.5%)
Number of joints involved	
Monoarthritis (1 joint)	6 (37.5%)
Oligoarthritis (2–3 joints)	7 (43.8%)
Polyarthritis (≥ 4 joints)	3 (18.8%)
Distribution of joint involvement	
Size	
Small joints	3 (18.8%)
Large joints^a^	10 (62.5%)
Small and large joints	3 (18.8%)
Symmetry	
Symmetric	2 (12.5%)
Asymmetric	14 (87.5%)
Location	
Upper extremities	6 (37.5%)
Lower extremities	5 (31.3%)
Upper and lower extremities	5 (31.3%)
RF (*N*^b^ = 11)	1 (9.1%)
Anti-CCP antibody (*N*^b^ = 11)	0 (0.0%)
ANA (*N*^b^ = 11)	0 (0.0%)
Hata’s classification	
Type I	2 (12.5%)
Type IIA	1 (6.3%)
Type IIB	8 (50.0%)
Type III	1 (6.3%)
Type IV	1 (6.3%)
Type V	3 (18.8%)

Data presented as *n* (%) unless stated otherwise

*SD* standard deviation, *RF* rheumatoid factor, *CCP* cyclic citrullinated peptide, *ANA* antinuclear antibody

^a^Most commonly involved was the shoulder joint

^b^Patients with missing data excluded

### Vascular manifestations associated with arthritis

Logistic regression analysis was performed to evaluate vascular manifestations associated with arthritis in TAK patients (Table 5). On univariate analysis, ESR, CRP, and type IIB vessel involvement were significantly associated with arthritis (*p* = 0.007, *p* = 0.027, and *p* = 0.004, respectively). Type V vessel involvement was inversely associated with arthritis (*p* = 0.048). On multivariate analysis, ESR (*p* = 0.012) and type IIB (p = 0.007) remained significant.

**Table 5:** Vascular manifestations associated with arthritis in Takayasu arteritis

Characteristic	Unadjusted OR	95% CI	*P* value
Univariate analysis			
ESR	1.015	1.004–1.025	0.007*
CRP	1.123	1.013–1.245	0.027*
Claudication	0.687	0.275–1.718	0.422
Decreased pulse	0.816	0.342–1.946	0.646
BP difference > 10 mmHg	1.657	0.474–5.792	0.429
Bruit	1.096	0.459–2.613	0.837
Carotidynia	0.877	0.243–3.163	0.841
Common carotid artery	1.658	0.713–3.853	0.240
Subclavian artery	2.241	0.931–5.394	0.072
Ascending aorta	1.844	0.869–3.913	0.111
Aortic arch	1.567	0.745–3.297	0.236
Descending aorta	2.156	0.957–4.857	0.064
Abdominal aorta	0.486	0.221–1.072	0.074
Renal artery	1.126	0.459–2.761	0.795
Iliac artery	0.230	0.030–1.751	0.156
Pulmonary artery	0.495	0.112–2.186	0.354
Type I	0.707	0.259–1.930	0.498
Type IIA	1.117	0.313–3.994	0.865
Type IIB	3.160	1.448–6.897	0.004*
Type III	0.814	0.100–6.643	0.847
Type IV	1.490	0.169–-13.177	0.720
Type V	0.441	0.196–0.992	0.048*
Multivariate analysis			
ESR	1.014	1.003–1.025	0.012*
CRP	1.052	0.911–1.215	0.489
Type IIB	2.956	1.337–6.537	0.007*
Type V	0.634	0.243–1.652	0.351

*OR* odds ratio, *CI* confidence interval, *ESR* erythrocyte sedimentation rate, *CRP* C-reactive protein, *BP* blood pressure

*Value considered significant (*p* < 0.05)

## Discussion

In this study, we determined that extravascular manifestations of TAK were not rare, with a prevalence of 19.0%. In particular, arthritis (11.9%) was the most common extravascular manifestation. To the best of our knowledge, this is the first study describing the extravascular manifestations of TAK in detail.

Sacroiliitis was present in 7.1% TAK patients. Considering that the global prevalence of SpA is approximately 1% [25], the proportion of TAK patients with sacroiliitis appears high. Although not clear to date, there might be a shared genetic background between TAK and sacroiliitis, which could play a central role in their co-occurrence. Further study regarding this issue could aid in elucidating the pathophysiologic background of these diseases. We discovered that the characteristics of sacroiliitis in TAK differed from SpA patients in general, being predominantly female (89.5%) and exhibiting a low incidence of HLA-B27 positivity (14.3%) [25–27]. This finding is consistent with a previous study [15] that also demonstrated female predominance and low HLA-B27 positivity. Furthermore, the presence of SpA features other than sacroiliitis in these patients was relatively low (peripheral arthritis 15.8%, enthesitis 5.3%, uveitis 5.3%, IBD 5.3%) compared to SpA patients in general (peripheral arthritis 39.8–58.0%, enthesitis 37.8–50.0%, uveitis 8.5–27.0%, IBD 1.8–11.0%) [28]. Considering that aortitis is one of the cardiovascular manifestations observed in ankylosing spondylitis and other forms of SpA [29], it can be challenging to differentiate TAK patients with sacroiliitis from SpA patients with aortitis. The aforementioned differences in the characteristics of sacroiliitis between these two patient types could provide clues in distinguishing one from another.

Peripheral arthritis was observed in 6.0% of TAK patients. This manifestation was characterized by an asymmetric oligoarthritis pattern, mostly involving the large joints. This was similar to the pattern observed in SpA (asymmetric oligoarthritis with lower extremity predominance) [30] except that an even distribution between the upper and lower extremities was observed.

Interestingly, the most common type of vessel involvement in TAK patients with arthritis (axial and/or peripheral arthritis) was type IIB, differing from the total study population where type V was most frequently observed. Furthermore, in the multivariate logistic regression analysis evaluating the association between arthritis and vascular manifestations, type IIB was associated with arthritis approximately three times higher than nontype IIB. Therefore, TAK patients with type IIB should be carefully monitored for arthritis.

In our study, the prevalence of IBD (2.6%) in TAK patients appeared low compared to previous reports (5.8–8.3%) [16, 31]. However, considering that the prevalence of CD and UC are 0.01% and 0.03%, respectively, in the general Korean population [32], a prevalence of 2.6% IBD in our TAK patients is obviously higher than that of the general population. This is consistent with a previous study reporting a higher prevalence of IBD in TAK patients than that in the general population [16, 31].

Recurrent oral ulcers (8.6%) were the second most common extravascular manifestation in our study, whereas erythema nodosum (1.5%) and uveitis (0.7%) were rarely observed. Recurrent oral ulcers, erythema nodosum, and monoarthritis or oligoarthritis are manifestations that are also observed in Behcet’s disease, as well as inflammation of large vessels [33]. Thus, it may be confusing to distinguish TAK from Behcet’s disease. However, a notable difference between TAK and Behcet’s disease is the absence of genital ulcers. In our data, none of the TAK patients exhibited genital ulcers, which have the highest discriminatory value in the International Study Group for Behcet’s Disease criteria [34]. Furthermore, occlusion or stenosis due to homogeneous concentric wall thickness favors TAK, whereas thrombotic occlusion or a solitary aneurysm is more likely in Behcet’s disease [33].

The present study has some limitations. First, this study was retrospective. Although not many, there were some missing data concerning clinical symptoms and signs. Therefore, there might be other extravascular manifestations that we have not identified. Second, only Korean TAK patients were included, and other ethnic populations could display different results. Thus, further studies with subjects from different ethnic populations are required. Despite these limitations, considering that TAK is such a rare disease, our results are strengthened by the relatively large number of patients.

## Conclusions

In summary, we have shown that extravascular manifestations of TAK are observed in up to one-fifth of TAK patients. The most common extravascular manifestation was arthritis (axial arthritis and peripheral arthritis), and Hata’s type IIB pattern of vessel involvement and a high ESR were associated with the presence of arthritis. The presented results are meaningful in that they provide clinicians with a better understanding of the accompanying extravascular manifestations of this rare disease.
